# Establishment of Patient-Derived Xenotransplant and Cell Line Models for Canine B-Cell Lymphoma

**DOI:** 10.3390/cells15181684

**Published:** 2026-09-17

**Authors:** Wilfred Leung, Timothy M. Pierpont, Suzin Webb, Jessica J. Hayward, Shirley Markant, Nicole Belcher, Scott D. Butler, Rishi Puri, Michael J. Byron, Marta G. Castelhano, Giorgio Inghirami, Andrew D. Miller, Tracy Stokol, Kristy L. Richards, Angela L. McCleary-Wheeler, Robert S. Weiss

**Affiliations:** 1 Department of Biomedical & Translational Sciences, College of Veterinary Medicine, Cornell University, Ithaca, NY 14853, USA; wl475@cornell.edu (W.L.); tmp68@cornell.edu (T.M.P.); smw266@cornell.edu (S.W.); jjh276@cornell.edu (J.J.H.);; 2 Department of Clinical Sciences, College of Veterinary Medicine, Cornell University, Ithaca, NY 14853, USA; slm30@cornell.edu (S.M.); mgc27@cornell.edu (M.G.C.); 3 Department of Population Medicine and Diagnostic Sciences, College of Veterinary Medicine, Cornell University, Ithaca, NY 14853, USA; nb87@cornell.edu (N.B.); andrew.miller@cornell.edu (A.D.M.); ts23@cornell.edu (T.S.); 4 PATh PDX/CDX and Surgical Facility, Department of Biomedical & Translational Sciences, College of Veterinary Medicine, Cornell University, Ithaca, NY 14853, USA; scott.butler@colostate.edu (S.D.B.); rp422@cornell.edu (R.P.); 5 Clinical Sciences Innovation Laboratory, Department of Clinical Sciences, College of Veterinary Medicine, Cornell University, Ithaca, NY 14853, USA; mb2674@cornell.edu; 6 Cornell Veterinary Biobank, College of Veterinary Medicine, Cornell University, Ithaca, NY 14853, USA; 7 Pathology and Laboratory Medicine, New York Presbyterian Hospital, Weill Cornell Medicine, New York, NY 10065, USA; ggi9001@med.cornell.edu; 8 Department of Medicine, Sandra and Edward Meyer Cancer Center, Weill Cornell Medicine, New York, NY 10065, USA

**Keywords:** canine B-cell lymphoma, cBCL, patient-derived xenograft, PDX, diffuse large B-cell lymphoma, DLBCL, comparative oncology

## Abstract

**Highlights:**

**What are the main findings?**

• Three novel patient-derived xenograft (PDX) models and two stable cell lines were successfully established from spontaneous canine B-cell lymphoma tumors.
• Comprehensive characterization indicated that the genetic and physiological features of these models remained consistent with those of the original patient tumors even after serial transplantation.

**What are the implications of the main findings?**

• These validated models provide essential new tools for testing therapies, addressing the scarcity of laboratory resources in canine lymphoma research.
• Given the similarities between canine B-cell lymphoma and human non-Hodgkin lymphoma, these resources support comparative oncology studies that may benefit both veterinary and human medicine.

**Abstract:**

High-grade canine B-cell lymphoma (cBCL) is a spontaneous cancer in dogs with similar pathologic features as human diffuse large B-cell lymphoma (DLBCL). The value of comparative oncology to both dogs and humans has long been recognized, but the progress in cBCL research has been hampered by a scarcity of preclinical models. This study aimed to establish and characterize novel patient-derived xenograft (PDX) and cell line models from primary cBCL tumors to address this critical gap. To this end, primary cBCL tumors from three canine patients were surgically excised and serially transplanted into immunodeficient NOD scid gamma (NSG) mice. The resulting PDX models were characterized across passages in mice using histology, flow cytometry, single-nucleotide polymorphism (SNP) genotyping, and RNA sequencing. The results show that the samples retain many of their original characteristics, even after multiple NSG passages. Cancer cells from two of the PDX lines were successfully adapted to in vitro culture, creating two new established cell lines which were then used in drug sensitivity assays. The successful generation and characterization of these cBCL PDX and cell lines provides a valuable resource for canine cancer research and associated comparative oncology approaches.

## 1. Introduction

Non-Hodgkin lymphoma (NHL) is the most common hematopoietic malignancy found in humans, with over 80,000 new cases diagnosed each year in the USA [1]. Diffuse large B-cell lymphoma (DLBCL) is the most common subtype of NHL, accounting for 40% of all cases. Standard of care treatment is an intensive immunochemotherapy regimen of R-CHOP (rituximab, cyclophosphamide, doxorubicin, vincristine, prednisolone), resulting in a 60% cure rate [2]. Gene expression profiling reveals two distinct cell-of-origin subtypes, termed germinal center B-cell like (GCB) and activated B-cell like (ABC) DLBCL, with the latter associated with unfavorable survival outcomes [3]. Recent genomic studies reveal a vast genetic heterogeneity with hundreds of putative driver mutations identified and further classified DLBCL into distinct subtypes based on mutations, translocations, and copy number alterations [4,5,6]. These genomic subtypes are associated with survival and may be a guide for precision medicine approaches targeting specific genetic alterations enriched for each subtype [7]. Genetically engineered murine models recapitulate certain pathological features observed in human DLBCL, but lack the genetic diversity and immune microenvironment observed in the patient population [8,9]. This limitation of immunodeficient mouse models is especially pertinent given the new generation of therapies, including CAR-T and T-cell-engaging bispecific antibodies, which rely on the immune system to initiate tumor cytotoxicity [10,11].

B-cell lymphomas, which arise spontaneously in pet dogs, can be used as a naturally occurring model of DLBCL in humans due to shared clinical and pathological attributes [12]. Although canine B-cell lymphomas (cBCL) do not share identical mutations with their human counterparts, they do have similar signaling, proliferation, and survival pathways, suggesting a common pathogenesis [13,14]. For example, TNF receptor-associated factor (TRAF3), a negative regulator of nuclear factor NF-κB, is the most common mutated protein in cBCL and is also mutated in human DLBCL, evidence that the oncogenic NF-κB signaling pathway is important for lymphoma development in both species [15]. Epigenetic modifying factors are the most common class of mutations found in human DLBCL. Mutations in histone *H3C8* (resulting in H3K27M alteration) and histone methyl demethylase *KDM6A* both impact lysine residue 27 of histone 3 and are commonly found in cBCL [16]. Similarly, the second most frequent mutation in cBCL is the histone methyltransferase gene, *SET domain containing 2* (*SETD2*), which has been found to induce a hypermutator state in human DLBCL [17,18]. Dogs retain an intact immune system and are exposed to the same environments as humans, making them an ideal animal model for concurrent drug discovery [19]. The Bruton tyrosine kinase inhibitor, ibrutinib, emphasizes this valuable connection, as it is currently being used to treat human B cell malignancies, but was first shown to have efficacy in cBCL before it was approved for use in humans [20,21].

Although progress has been made in our understanding of the shared genetic and tumor biology of canine and human B-cell lymphoma, there is a substantial lack of reagents, cell lines, and models for cBCL. Lymphoma cell-line generation from primary samples is difficult, and protocols to generate stable cell lines do not exist. Currently, there are only two commonly available canine malignant B-cell cell lines, 1771 and CLBL1 [22,23]. This is in contrast to the hundreds of human lymphoma cell lines that are readily available, extensively characterized, and play invaluable roles in research and drug development [24]. Also worth noting is that clinical outcomes for cBCL are poor, and there remains a pressing need for new therapies in dogs. Although standard of care CHOP (cyclophosphamide, hydroxydaunorubicin [doxorubicin], oncovin [vincristine], and prednisone) chemotherapy is effective in inducing remission in the majority of canine patients, the disease almost invariably recurs and median survival time is only 12 months [25]. To help accelerate our understanding of cBCL biology and the development of targeted therapies, we established a protocol in which cBCL cells are extracted by lymph node biopsy and xenografted directly into immunodeficient NOD scid gamma (NSG) mice. Three independent cBCLs were successfully expanded as patient-derived xenografts (PDXs) in NSG mice, allowing for propagation of tumor cells and study of tumor evolution using histology and immunohistochemistry, flow cytometry, genotyping (DNA sequencing for single-nucleotide polymorphisms [SNPs] and diversity of the variable region of the immunoglobin heavy chain gene [IgHV]), and bulk RNA sequencing. Transplantation into mice allowed for the selection of tumor clones that subsequently grew in vitro as stable cell lines for two of the cBCLs, which we named KLR-1906S and KLR-1201, derived from the PDX lines named 15810-1906 and 19419-1201, respectively. These cell lines showed genetic and phenotypic features resembling those of the original tumor and allowed for drug treatment and genetic manipulation using shRNA and CRISPR-Cas9. Generation of these new cBCLs as transplantable patient-derived xenografts in mice, and subsequently as established cell lines, expands our knowledge of cBCL and provides a new platform where drug combinations that may benefit humans and dogs can be tested in vitro and in vivo.

## 2. Materials and Methods

### 2.1. Dog Patient History and Clinical Diagnosis

Peripheral lymph node tissue samples were taken from three dogs diagnosed with B-cell lymphoma at the Cornell University Companion Animal Hospital (Table 1). Whole lymph node extirpation for sample collection was approved under IACUC protocol #2005-0151 (Cornell Veterinary Biobank) and performed following informed owner consent. All dogs had been newly diagnosed with lymphoma and had not received any prior therapy. B-cell lymphoma was verified by a board-certified pathologist via histopathologic analysis and immunostaining for clinical B-cell markers (e.g., Pax-5).

### 2.2. Histology and Immunohistochemical Staining

All tissues were fixed in 10% neutral buffered formalin with processing, sectioning, and hematoxylin and eosin (H&E) staining performed following standard laboratory procedures in the histopathology laboratory in the Animal Health Diagnostic Center at Cornell University. In addition to H&E staining, sections were processed for immunohistochemistry using a DAB-based detection system to visualize CD20 (B-cell marker) and CD3 (T-cell marker) expression [26].

### 2.3. Patient-Derived Xenograft Generation

Generation of canine PDX was performed by the Cornell College of Veterinary Medicine PATh PDX/CDX Facility. Primary tumor samples were collected either during surgical excision or through rapid postmortem tissue collection. Soon after excision, tumor tissue was sliced into roughly 1 mm^3^ pieces under sterile conditions, while submerged in a small amount of fetal bovine serum (FBS) before being transported to the mouse facilities. The study utilized NOD scid gamma (NSG) immunocompromised mice, housed in an ABSL2 barrier facility. Animals were housed socially in microisolator cages, kept on a 14/10 light dark cycle and given food and water ad libitum. NSG mice were anesthetized using isoflurane, and the minced tumor tissue along with 100 µL of media was placed into a syringe and injected with an 18-gauge needle subcutaneously into the flanks of the mice. Mice were monitored several times a week for signs of successful tumor implantation by physical palpation. For comparative analysis of drug sensitivity, a human refractory DLBCL PDX model from a 41-year-old Caucasian male who underwent DICE regimen (DLBCL_p3_NY-DB-TE) was evaluated concurrently. Subcutaneous implantation in mice is generally well tolerated; however, animals showing pain or distress post-implantation would be provided ketoprofen every 24 h up to 48 h post-procedure.

### 2.4. Cell Line Generation and Growth Analysis

cBCL cell lines were established from PDX specimens 15810-1906 and 19419-1201 (the last 4 digits indicate passage 19 and 12, respectively, as well as the specific NSG mouse). Tumor tissue was excised from PDX mice and transferred into sterile DMEM media (Gibco) containing 10% FBS (Gibco) and Penicillin/Streptomycin (Gibco). Tissue was dissociated by manual separation on ice and passed through a 70 µm filter to create a single cell suspension. Cells were washed in sterile PBS, resuspended in sterile IMDM media (Gibco) with 20% FBS, and initially seeded at 1–5 × 10^6^ cells/mL. All cells were grown in a 37 °C incubator in a humidified atmosphere of 5% CO2. Fresh media was added once per week. Once logarithmic growth of cells was observed, the cell line was expanded and frozen down in freezing media (10% DMSO and 90% FBS) for storage in liquid nitrogen. Cell lines were maintained in IMDM media with 20% FBS and seeded at 0.5–1 × 10^6^ cells/mL with media changes every 2–3 days.

### 2.5. Genotyping Assays

#### 2.5.1. Single-Nucleotide Polymorphism-Based Fingerprinting

DNA from the primary tumors, PDX, and KLR-1906S and KLR-1201 cell lines, as well as DNA from the established cell lines 1771 and CLBL1, were genotyped on the semi-custom CanineHD 220k beadchip (Illumina, CA, USA) (Table 2) [23,27].

Quality control of the genotype data was performed in PLINK 1.9 [28]. Single-nucleotide polymorphisms (SNPs) were removed based on a genotyping rate of <95%, a minor allele frequency (MAF) of <5%, and discordance between duplicate samples from a previous study (*n* = 752 SNPs) [29]. After filtering there were 135,284 SNPs remaining.

The data were pruned to remove linked SNPs (using PLINK’s --indep 50 5 2 command), leaving 5277 SNPs for an identity by descent (IBD) calculation. This was performed in PLINK, using a metric called pi-hat, which is a measure of the probability that two samples share 0, 1 or 2 alleles. From the 5277 SNPs, a further 106 non-autosomal SNPs were excluded from the IBD analysis. All 9 samples were included, and pi-hat was calculated for each pair.

A genotyping fingerprint was created by selecting 94 SNPs across the genome to distinguish the B-cell lymphoma cell lines from each other. Criteria for selection of the 94 SNPs included: a total of between 50 and 100 SNPs genome-wide, missingness < 1%, MAF ≥ 30% in the 9 samples, located at least 5 Mb apart, 2–3 SNPs per chromosome including chromosome X, and exclusion of mitochondrial and Y chromosome SNPs. Further selection was based on SNPs where the 19419-sample group (*n* = 4), 15810-sample group (*n* = 3) and either CLBL1 or 1771 each have a different genotype. All 94 SNPs except two were ≥10 Mb apart.

To show the relationship between each of the 9 samples for these 94 SNPs, a heatmap was generated using the package pheatmap v 1.0.2 [30] in R version 4.0.2. In order to determine the uniqueness of the SNPs chosen for fingerprinting, pairwise IBD was calculated using only the 94 fingerprint SNPs for all 9 samples in this study (15810, 19419, 1771, CLBL1) and a further 5981 dogs that have been previously genotyped [29].

#### 2.5.2. Sequencing and Analysis of the Variable Region of the IgH Gene

Genomic DNA was prepared from flash-frozen PDX passaged tumors and fresh cell lines (KLR-1906S and KLR-1201) using standard salt DNA extraction, as well as cell-free DNA from plasma of relevant PDX mice (QIAamp Circulating Nucleic Acid Kit, QIAGEN, Venlo, The Netherlands). Genomic DNA samples were quantified on a Nanodrop (Thermo Scientific, Waltham, MA, USA), while cell-free DNA samples were quantified on a QUBIT fluorometer (Life Technologies, Carlsbad, CA, USA) using the dsDNA High-Sensitivity Assay Kit (Invitrogen, Waltham, MA, USA) due to the expected low-yielding, highly fragmented DNA typical of cell-free DNA [31]. Genomic DNA samples were diluted to 25 ng/µL, while cell-free DNA samples were used undiluted as templates for PCR. Primers for PCR yielded an approximately 600 bp product and were IGHV.NGS.F1 (5′-CCATGGAGTCTGTGCTCTGC) and IGHV.NGS.R2 (5′-CTGAGGAGACGGTGACCAGG) [25]. PCR was performed on a 9700 thermal cycler (Applied Biosystems, Waltham, MA, USA) using the following cycling conditions: 95 °C for 10 min, 40 cycles of 95 °C for 30 s/59.5 °C for 30 s/72 °C for 1 min, 72 °C for 5 min, and a final 4 °C hold. PCR products were cleaned up using AMPure XP Beads (Beckman Coulter, Brea, CA, USA) and then a 1 ng/uL (or undiluted) aliquot was submitted to the Cornell Institute of Biotechnology Genomics Facility for pooling and sequencing on Illumina MiSeq (2 × 300 bp).

Primer sequences and very low-quality 3′ bases (Phred score < 10) were trimmed from the raw fastq reads, and any reads <240 bp were removed, using cutadapt 2.10 [32]. Trimmed sequences were run through FastQC [33] using multiQC [34] to visualize the output. The forward and reverse paired-end reads were assembled using PEAR v0.9.6 [35]; the outputs were converted to fasta using seqtk v1.2-r102-dirty [36] and then aligned to canine IGH sequences using the international ImMunoGeneTics information system (IMGT) website http://imgt.org/HighV-QUEST/ (accessed on 15 February 2023).

Aligned sequence outputs in the form of summary.txt files were run through a Jupyter notebook [37] to filter out the productive sequences and create a file of sequence counts for each V-region subtype. Stacked bar graphs showing the percentages of sequences aligning to each subtype for each sample were created in R.

### 2.6. Flow Cytometry Analysis of Primary Tumors, PDX and PDX-Derived Cell Lines

Fine-needle aspirates on primary tumors in the peripheral lymph nodes of two dogs (19419 and 14298) were placed into media (DMEM with 20% FBS) for flow cytometric analysis, which was performed on the day of aspiration. After centrifugation to remove excess media (500× *g* for 5 min at 4 °C), red blood cells were lysed in 3 mL of ammonium chloride buffer (150 mM ammonium chloride, 1.2 mM EDTA, 10 mM sodium bicarbonate) for 3–5 min, followed by washing and resuspension in PBS, supplemented with 0.1% BSA (Sigma-Aldrich, for all reagents other than those specified) and 0.05% sodium azide. Fluorescent-conjugated antibodies against various leukocyte-associated markers were then added to cells (0.1–1 × 10^6^) for 30 min on ice. For samples with conjugated antibodies, the cells were washed in PBSA and resuspended in PBS for analysis. For unconjugated antibodies, a 30 min incubation on ice with a fluorescent-conjugated secondary antibody diluted in PBSA (1:200) was added after the first PBSA wash, followed by a final PBSA wash and resuspension in PBS. The cells were then analyzed for antibody binding using a flow cytometer (FACSCalibur™, BD Biosciences, San Jose, CA, USA) as described [38]. Similar lysis and staining procedures were used for cells derived from mouse xenografts, whereas the lysis procedure was not required for cultured cells, which were suspended directly in PBSA after centrifugation.

Targeted antigens (and their corresponding registry numbers) used for these experiments are as follows: CD4 (Bio-Rad Cat# MCA1038F, RRID: AB_321271, Hercules, CA, USA), CD8 (Bio-Rad Cat# MCA1039PE, RRID: AB_322646), MHCII (Bio-Rad Cat# MCA1044F, RRID: AB_322642), CD34 (BD Biosciences Cat# 559369, RRID: AB_397238), CD3 (Bio-Rad Cat# MCA1774F, RRID: AB_2291174), CD5 (Bio-Rad Cat# MCA1037F, RRID: AB_322643), CD45 (Bio-Rad Cat# MCA1042F, RRID: AB_324047), CD21 (BD Biosciences Cat# 555422, RRID: AB_395816), CD22 (Abcam Cat# ab23620 RRID: AB_447570, Cambridge, UK), CD14 (Agilent Cat#R086401, RRID: AB_579551, Santa Clara, CA, USA), CD25 (Thermo Fisher Scientific Cat# 50-0250-42, RRID:AB_10609350), CD28 (Thermo Fisher Scientific Cat# 17-0282-42, RRID:AB_2573140), CD90 (clone CA1.4G8, Peter Moore, University of California-Davis, Davis, CA, USA) and CD18 (Bio-Rad Cat#MCA1780, RRID:AB_2128639).

### 2.7. Bulk RNA Sequencing of Primary Tumors and PDX and Transcriptional Analysis

Total RNA was isolated from flash-frozen primary canine tumor and PDX tissue using Trizol extraction. Library preparation, sequencing, and initial data processing were performed by the Transcriptional Regulation & Expression (TREx) Facility at Cornell University. Libraries were generated using the NEBNext Ultra II Directional RNA kit with polyA enrichment, utilizing 750 ng of total RNA as input. Sequencing was conducted on an Illumina NextSeq 500 platform, producing 85 bp single-end (SE) reads. Raw reads were processed using Cutadapt (v1.8.3) for 3’ adapter removal and quality trimming, followed by alignment to the Canis lupus familiaris reference genome and transcriptome (CanFam3, Ensembl annotations) using TopHat (v2.1.1). Fragments Per Kilobase of transcript per Million mapped reads (FPKM) values were generated using Cufflinks (v2.2.1).

Unexpressed genes were filtered out of the resulting FPKM dataset. Integrated Differential Expression & Pathway analysis (iDEP) version 2.01 [39] was used for the clustering of genes based on their expression patterns across all samples. We employed the k-Means algorithm, using the top 2000 genes exhibiting the most variable expression. The samples were assembled, and the genes were clustered into 5 distinct groups. Following the identification of gene clusters, enrichment analysis was conducted for each cluster utilizing gene ontology cellular components for all 5 resulting clusters. Principal component analysis (PCA) was conducted using PC1 and PC2.

Differentially expressed genes between primary and PDX tumors were identified as having a minimum 2-fold difference with a 0.1 false discovery rate cutoff.

### 2.8. Gene Knockdown by ShRNA

Parental cells were transduced with lentivirus expressing shRNA targeting KDM6a (KDM6A sh#1: 5′ GCC TCA GGA TGC CAT TAA A 3′) and puromycin resistance gene in vector pGreenPuro (SBI #SI505A-1) followed by 7 days of puromycin selection (1 ug/mL for 19419, 0.5 ug/mL for 15810). Knockdown was verified by Western Blot.

### 2.9. Gene Editing with CRISPR-Cas9

Parental cells were transduced with lentivirus expressing doxycycline inducible Cas9 and blasticidin resistance gene (Addgene, #83481) followed by 5 days of blasticidin selection (20 ug/mL for 19419, 6 ug/mL for 15810). Cas9 expressing cells were transduced with lentivirus expressing a KDM6a specific sgRNA (KDM6AG#1: 5′ ACT CCA GGC TGA GAG ACG CT 3′) and GFP (Addgene, #57822). Four days post-doxycycline (1 ug/mL) induction, GFP+ cells were single-cell sorted into 96 well plates containing 20% FBS, 20% media from parental cell line passed through a 0.45 um filter and 60% IMDM and allowed to grow for at least 2 weeks. Clones were screened by PCR amplification of a 500 bp region encompassing the CRISPR-Cas9 cleavage site and verified by Sanger sequencing at the Cornell Institute of Biotechnology Genomics Facility. Cas9 expression and CRISPR-Cas9-mediated loss of KDM6a expression was additionally verified by Western blot.

### 2.10. Western Blot

Protein lysates were prepared in RIPA buffer (ThermoFisher) and lysed by vortexing. Protein concentration was determined using a BCA protein assay kit (ThermoFisher #23225) with a BSA standard curve. Samples were resolved by SDS-polyacrylamide gel electrophoresis (Biorad), transferred to a PVDF membrane (ThermoFisher) and probed with primary antibody KDM6a (ThermoFisher # PA5-31828) or FLAG (Sigma #F1804) followed by HRP-conjugated secondary antibody and signal determined by chemiluminescence (Biorad).

### 2.11. Survival Curve and Statistical Analysis

Kaplan–Meier survival curves for recipient NSG mice were generated in R (v4.3.3) using ggsurvplot from the survminer package. For each of the three PDX lines, time-to-event was defined as the duration in days from subcutaneous tumor injection to the date the tumor-size humane endpoint (1.125 cm^3^) was reached. Mice that died or needed to be euthanized due to causes unrelated to tumor progression were censored at the time of death.

Statistical analysis was performed using R. To provide a clear visualization of survival trends, and to account for lower numbers in individual passages, passage numbers were grouped: P 1–4, P 5–9, P 10–14, P 15–19, and P 20–24. Differences between survival distributions across all passage groups were evaluated using a global log-rank test.

### 2.12. In Vitro Drug Response

Cells were seeded into a 96 well plate at 0.4 × 10^6^ cells/mL, in triplicate per dose. Doxorubicin hydrochloride (Sigma #D1515) was added to cells, followed by incubation at 37 °C in a humidified environment supplemented with 5% CO2. Alamar blue (ThermoFisher) was added after 46 h and fluorescence read at 46 and 48 h with the Synergy NEO microplate reader (BioTek). The cell numbers of viable drug-treated cells were normalized to vehicle treated controls and expressed as percentage cell number. Experiments were performed in triplicate.

### 2.13. Pilot Study of Chemotherapeutic Response in Mice Bearing Canine B-Cell PDX

As a proof-of-concept study, PDX lines 15810 (passage 8) and 19419 (passage 11) were transplanted into approximately 6-week-old female NSG mice, *n* = 1 per xenograft, as described above and allowed to grow until at least one dimension was 20 mm, measured using calipers. CHOP treatment groups were injected intravenously on the morning of day 1 with cyclophosphamide (30 mg/kg IV, Sigma #C3250000), doxorubicin hydrochloride (2.48 mg/kg IV, Sigma #D1515), vincristine sulfate (0.38 mg/kg IV, Sigma, #V8879) and with prednisone (0.02 mg/kg IM, Sigma #D6004) intramuscularly in the afternoons on days 1–5. Tumor burden was monitored twice per week, as primary outcome measure of experimental treatment by vernier caliper, estimating tumor volume using the formula (LxW^2)/2. Mice were monitored until meeting humane endpoint criteria, including severe lethargy, loss of more than 20% body weight, difficulty breathing, excessive tumor burden affecting ambulation or tumor ulceration in accordance with approved Cornell University IACUC protocol #2017-0035. Study participants were not blinded as all subjects underwent the same experimental treatment.

## 3. Results

### 3.1. Primary Tumor Collection and PDX Generation

#### 3.1.1. Primary Tumor Characterization

**Figure 1:**
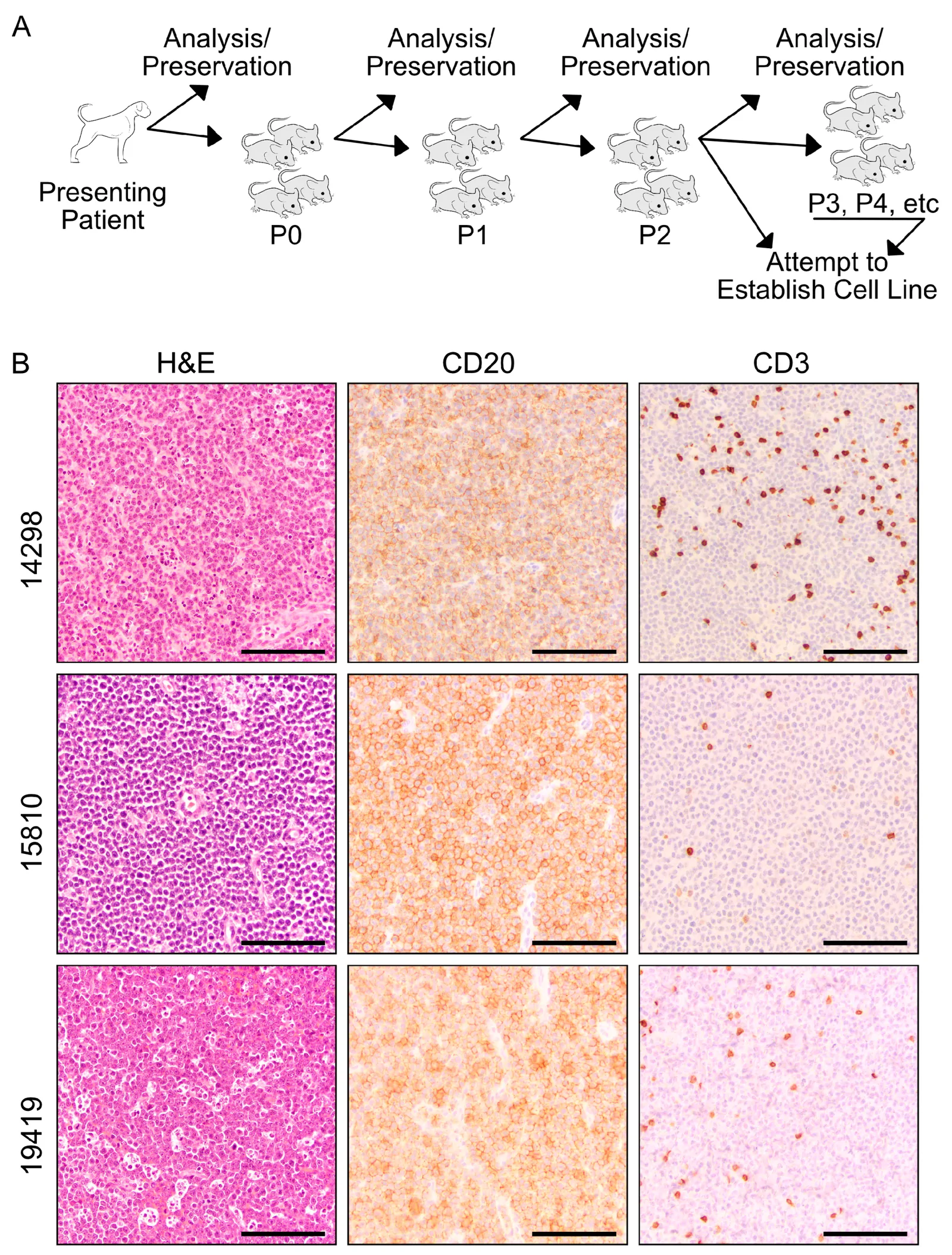
Primary lymphoma samples from canine patients. (**A**) Schematic showing the general workflow from initial excision to propagation and finally establishment of cell lines. (**B**) Histologic sections from primary patient tumors stained with hematoxylin and eosin (H&E) and DAB-based immunohistochemical (IHC) labeling for CD20 (B-lymphocyte marker) and CD3 (T-lymphocyte marker). Scale bars indicate 100 µm.

A total of three canine patient tumors from peripheral lymph node excisional biopsies were propagated in NSG mice to generate murine xenografts (Figure 1A). Examination of H&E-stained sections of formalin-fixed paraffin-embedded tissue showed effacement of lymph node architecture by intermediate to large round cells, with a large round to oval nucleus containing prominent nucleoli, and scant amounts of cytoplasm. Immunohistochemical staining for CD20 confirmed a B-cell origin for the tumor, with residual CD3+ T cells (Figure 1B).

#### 3.1.2. cBCL Patient-Derived Xenografts

Tumors were propagated by subcutaneous injection into immunodeficient NSG mice to sustain tumor cell growth and tumor microenvironment characteristics. Kaplan–Meier analysis showed a consistent trend across all three PDX models: serial passaging significantly shortened recipient mouse survival, indicating an increase in tumor aggressiveness and growth rate over time (*p* < 0.0001 for all).

**Figure 2:**
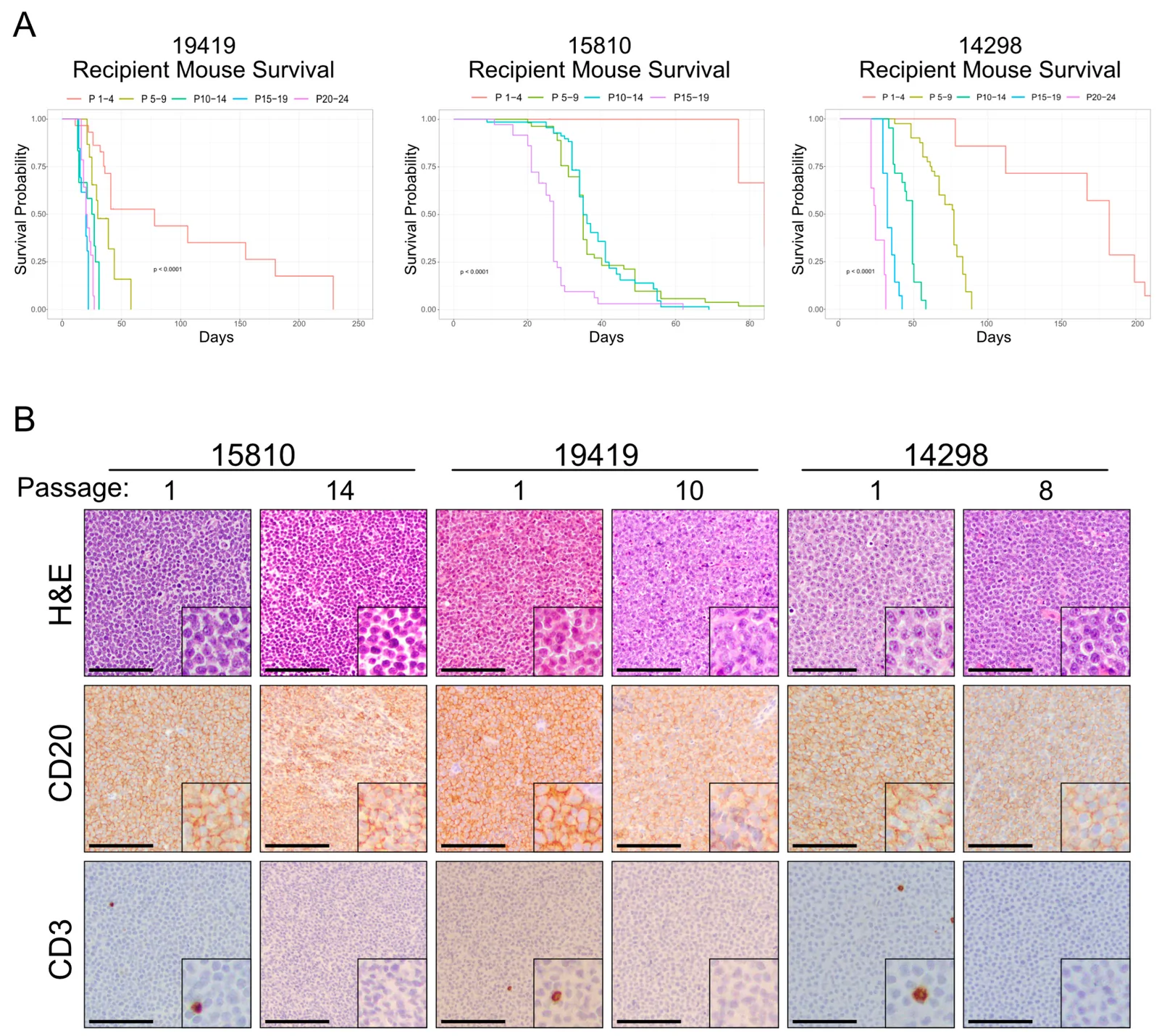
Survival and histopathological analysis of cBCL PDX in NSG mice. (**A**) Kaplan–Meier survival curves of NSG mice xenografted with serial passages of canine lymphoma PDX. Due to low sample sizes for individual passages, data were grouped into sequential blocks (P 1–4, P 5–9, P 10–14, P 15–19, and P 20–24) for analysis. *p*-values indicate differences between survival distributions across all passage groups as evaluated by a global log-rank test. (**B**) Histology of patient-derived xenograft tumors stained with hematoxylin and eosin (H&E), and DAB-based IHC labeling for CD20 and CD3, showing retention of features of the original tumor, which are CD20-positive with sparse CD3-positive cells. Scale bars indicate 100 µm. The magnified insets are at 2.5× magnification relative to the main images.

For all three PDX lines, mice in the earliest passage group (P 1–4) consistently exhibited the longest survival. With each successive block of passages, the xenografted mice reached humane endpoints based on tumor size in shorter times, indicating that the cancers became more aggressive with passaging (Figure 2A). The median times were 26, 34, and 55.5 days for 19419, 15810, and 14298, respectively. Sustained passaging was observed across all tumors that were successfully transplanted and grew from the primary canine tumor.

#### 3.1.3. Histologic and Immunophenotypic Features of the cBCL PDX

Analysis of H&E-stained sections of the PDX at two different passages in NSG mice revealed similar tissue architecture to primary tumor, containing sheets of large round cells, with round to oval nuclei and prominent nucleoli (Figure 2B). The tumors were well vascularized, likely supporting sustained tumor growth. In comparison to earlier passages, later passages of xenograft tissue showed more compact cells and coarse chromatin, consistent with faster proliferation timing and shorter time between passages. Expansion of tumor cells displaying B-cell lineage (CD20+) was consistent across all xenografts and observed in xenografts serially transplanted over 10 times. T cells were still present after the 1st passage, however, in lower numbers compared to the original tumor. These CD3+ cells were canine patient-derived, as NSG mice lack B and T cells. Immunohistochemical staining of later passage xenografts generally showed a lack of T cells (Figure 2B).

#### 3.1.4. Generation and Genetic Manipulation of PDX-Derived Cell Lines

**Figure 3:**
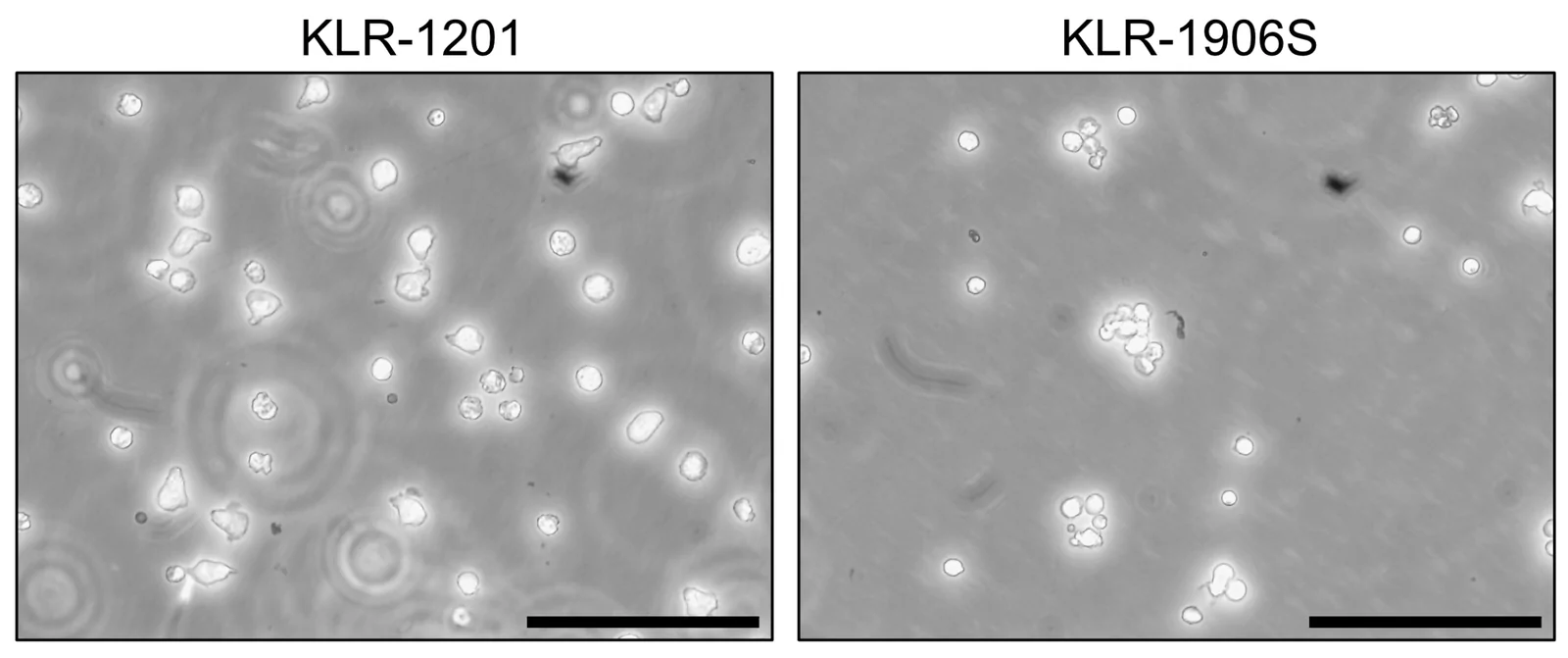
Bright-field images of cell lines KLR-1201 (**left**) and KLR-1906S (**right**) in culture, before passaging. Scale bars indicate 100 µm.

**Figure 4:**
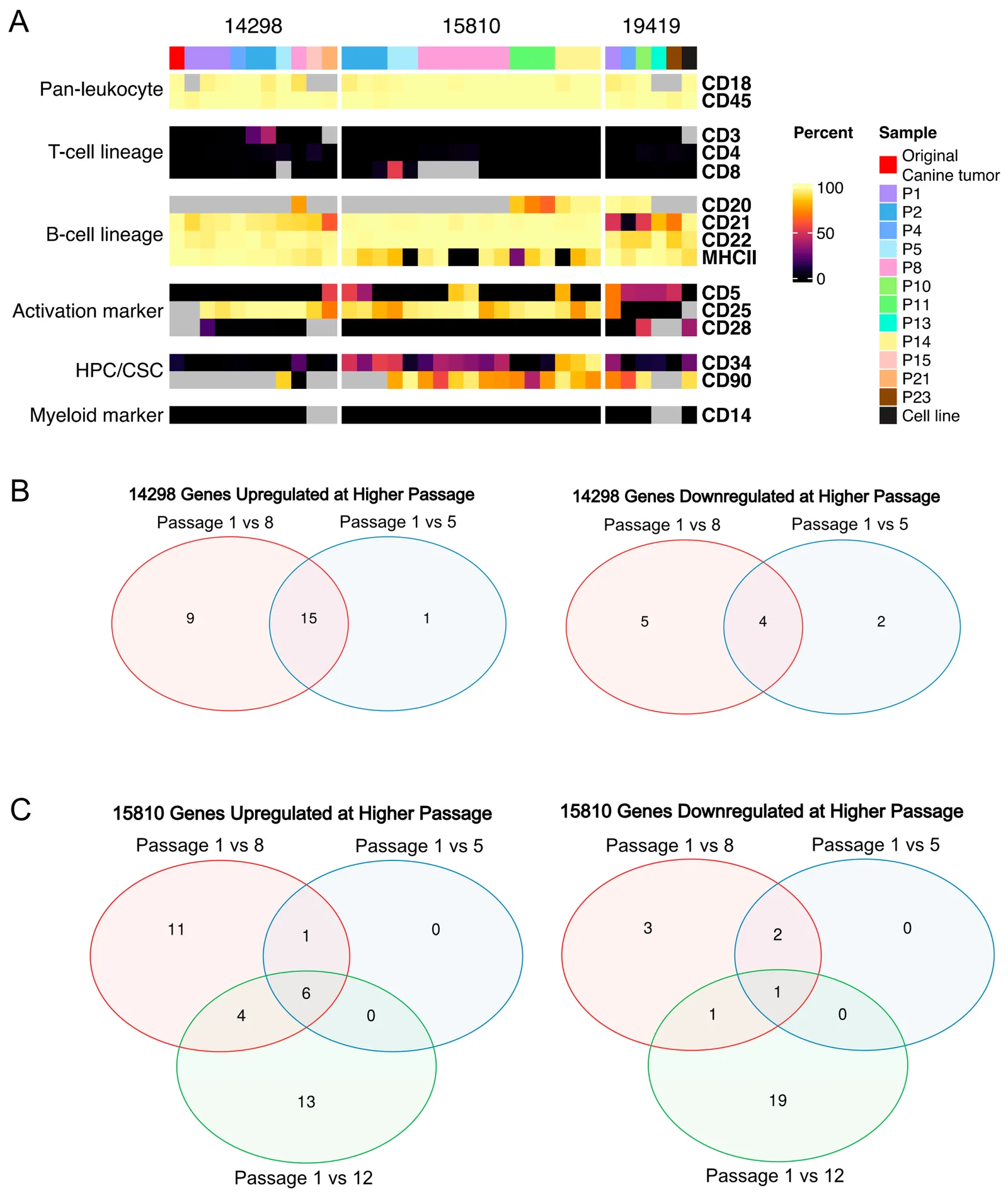
Surface protein and gene expression analysis of serially transplanted tumors. (**A**) Heatmap representing the percentage of cells with positive expression of cell surface markers by flow cytometry. (**B**) Venn diagrams show limited up- and down-regulation of differentially expressed genes between early and late passages of 14298. (**C**) Venn diagrams show limited up- and down-regulation of differentially expressed genes between early and late passages of 15810.

Following successful serial transplantation in mice, tumor cells from two of the PDXs were successfully cultured in vitro. The resulting B-cell lymphoma cell lines, designated KLR-1201 (from passage 26 from primary tumor 19419) and KLR-1906S (from passage 49 from primary tumor 15810) had characteristic round-cell morphology when analyzed by bright-field microscopy (Figure 3). On flow cytometric analysis, KLR-1201 retained antigen expression consistent with the first passage in NSG mice (results from the original tumor were not available), with the exception of loss of CD5 and gain of CD28 (Figure 4). The ability to study protein function by perturbing gene expression exemplifies the versatility of these cell lines. As a proof of principle for the ability to genetically manipulate these cell lines, KDM6a expression was perturbed by either shRNA knockdown or CRISPR-Cas9-mediated knockout in KLR-1201 cells, since KDM6a loss-of-function mutation is common in cBCL [16]. KDM6a protein levels were reduced in KDM6a shRNA cells and undetectable by Western blot following doxycycline induction and single-cell cloning of Cas9-CRISPR cells (Supplemental Figure S2A,B). CRISPR-Cas9-mediated insertion of a single-nucleotide insertion at the expected genetic site was confirmed by Sanger sequencing (Supplemental Figure S2C).

### 3.2. Genetic Characterization of Primary and PDX Tumors

#### 3.2.1. SNP Genotyping Assay

To characterize the genomic identity of the primary tumor and subsequent xenografts and cell lines, we genotyped DNA from these samples on the CanineHD beadchip. Identity by descent analysis, as calculated by the metric pi-hat, showed that samples from the respective primary tumors and PDX were all closely related (Table 3). For 19419, there was no difference (pi-hat = 1) between the two PDX samples (1201PDX and 1301PDX). Further, there was only a small difference (pi-hat = 0.9973 and 0.9978) between the 19419 primary tumor and the other 19419 samples, which corresponds to 11 SNPs of the 5171 included in the analysis. Nine of these 11 SNPs were heterozygous in the original tumor but homozygous (for the minor allele) in the PDXs and cell line, and the other 2 SNPs were homozygous in all the original and PDX samples but heterozygous in the cell line sample. Likewise, for 15810, there was only a small difference (pi-hat = 0.9948 and 0.9951) between the primary tumor and the PDX samples, which corresponds to 15 SNPs, 14 of which lost the heterozygosity present in the original tumor in the PDX and cell line samples, and one SNP that was homozygous in the primary and PDX samples but heterozygous in the cell line sample. IBD between 19419, 15810, and existing cell line 1771 and CLBL1 samples was non-existent (pi-hat = 0).

A heatmap created using the 94 SNPs selected for a lymphoma cell-line fingerprint for the 9 samples genotyped in the present study showed separate groupings for the 4 B-cell lymphomas, with all four 19419 and 15810 samples grouped together, respectively (Supplemental Figure S1). Calculated pairwise IBD (pi-hat measures) comparing the 94 fingerprint SNPs of the samples in the present study and a previously generated database of 6112 dogs showed that no other sample was closely related (all pi-hat < 0.55) (Supplemental Table S1). These data confirm the distinct genetic origins of these tumors, which were retained through serial passaging in NSG mice and in cell culture. No cross-contamination or divergence was observed over time.

#### 3.2.2. Flow Cytometric Analysis of Sequential PDX Passages

Expression of CD45, CD18, MHCII and B-cell lineage markers was consistent across PDX passages for all three PDX lines and with the primary tumors for the one cell line for which these data were available (14298) (Figure 4A). For 19419 and 14298, CD21 expression fluctuated between PDX passages. CD34 was weakly expressed in one original tumor (10% of cells) but was eventually lost with sequential xenografts (14298). In contrast, CD34 was expressed in most of the PDXs from the other two tumors (15810, 19419). Regarding T-cell markers, aberrant CD5 expression was variably observed in PDXs from two of the tumors (19419, 15810). Low percentages (2–7%) of CD4-positive cells were seen with increased passaging of PDX in all three tumors, one of which also had a subset of CD3+ cells (14298) (Figure 4A). Gain of T-cell markers in the PDX was attributed to the expansion of T cells, which can occur in immunocompromised mice or tumor heterogeneity [40]. On flow cytometric analysis, KLR-1201 retained antigen expression consistent with the first passage in NSG mice (results from the original tumor were not available), with the exception of loss of CD5 and gain of CD28 (Figure 4). Overall, our results indicate that PDX tumors and derived cell lines broadly maintained similar cell surface markers compared to the original tumor.

#### 3.2.3. Transcriptional Changes Across PDX Passages

To determine if transcriptional changes occurred over PDX passages, we identified differentially expressed genes by comparing the first to eighth passages of both 14298 and 15810 as well as by performing PCA of primary and PDX samples from all three tumors. Only a limited number of individual genes were differentially expressed (Figure 4B,C). Notably, minichromosome maintenance complex component 6 *(MCM6)*, which plays a critical role in cell cycle regulation, was down-regulated in the later passage of 14298, as was Polo-like kinase 4 *(PLK4)*, which plays an important role in centriole duplication. We also identified several differentially expressed genes in the 15810 PDX comparing passages 5 to 12, and 8 to 12, but no significant enrichment of pathways was identified using a KEGG analysis. Overall, these findings indicate that there is very limited change over time of these cBCLs when passaged in the NSG environment.

**Figure 5:**
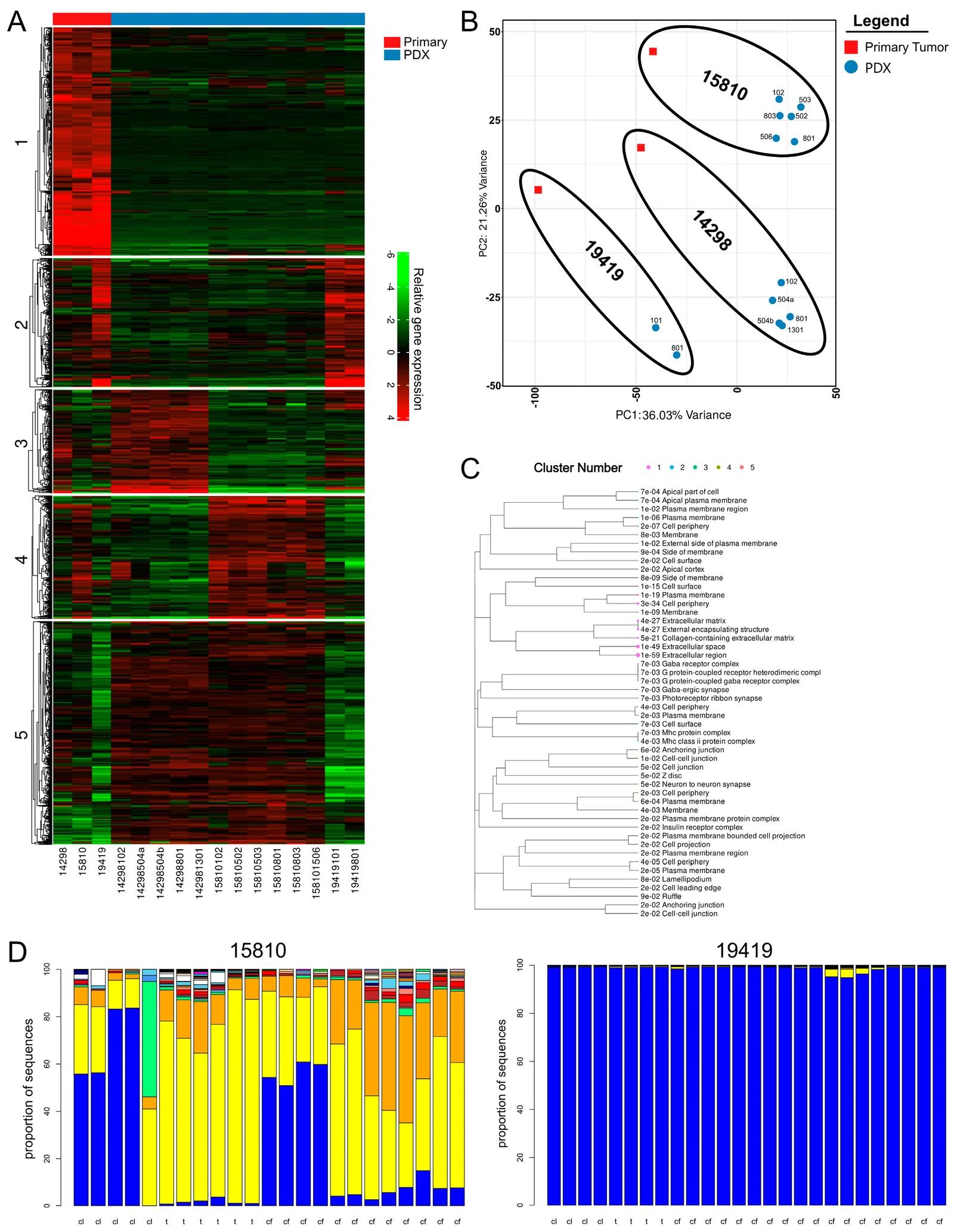
Gene expression comparison between primary and xenografted tumors. (**A**) Heatmap clustering of primary tumor and PDX samples based on the top 2000 most differentially expressed genes, broken into 5 clusters. (**B**) Principal component analysis of gene expression comparing primary and PDX tumors shows that the primary tumors are distinct, but cluster in line with their PDX counterparts. (**C**) Molecular pathways enriched in the 5 clusters identified in the heatmap, suggesting most of the pathways may be due to changes in extracellular spaces and plasma membranes. (**D**) IGHV sequence profiles from 15810 and 19419 samples, showing substantial diversity for the former and a predominantly single clonal sequence for the latter (cl = cell-line, cf = cell-free DNA, t = tumor). Colors represent different IGH subtypes.

As evidenced by the flow cytometric data, key diagnostic markers remained relatively unchanged after PDX passages compared to the primary tumors. We sought to identify unpredicted changes following the xenograft transplantation and found that analysis of samples using the top 2000 most differentially expressed genes caused primary tumors and PDX to cluster separately (Figure 5A). The PCA showed that PDX samples clustered by their origin rather than by passage number, with their respective primary tumor samples being distinct from the PDX specimens (Figure 5B). However, gene ontology pathway analysis of the main differences suggests they may primarily stem from changes related to changes in interactions with the extracellular environment of the cancer cells (Figure 5C). The observed changes could also be explained by the loss of canine cells that comprised the tumor microenvironment of the primary tumors, such as blood vessels and stromal and immune cells, which were replaced by mouse cells in the PDX environment. The murine genes were filtered out of this data set and thus host stromal and support cells are reflected in the RNA analysis of the primary tumors but not the PDXs in both the heatmap and PCA data. Thus, the data suggest that the PDX model may be most useful for testing cell cycle and other cancer cell intrinsic features.

#### 3.2.4. IGHV Sequence Diversity

Alignment of sequences to known canine IGH sequence subtypes show that 15810 samples exhibited a high degree of sequence diversity of the immunoglobulin heavy chain variable gene, as evidenced by a large number of IGH V-gene subtypes (Figure 5D). This pattern is consistent with multiple clonally independent malignant populations and/or ongoing somatic hypermutation, which has been documented in canine B-cell lymphoma samples [25]. In contrast, 19419 samples exhibited a high percentage (>95%) of a single clonal sequence (Figure 5D), displaying the signature of a single malignant clone. These results highlight the importance of creating multiple xenograft models for cBCL, as these subtypes may have different clinical outcomes or responses to therapy [25].

### 3.3. Cell Line and PDX Drug Sensitivity

#### In Vitro and in Vivo Drug Responses

**Figure 6:**
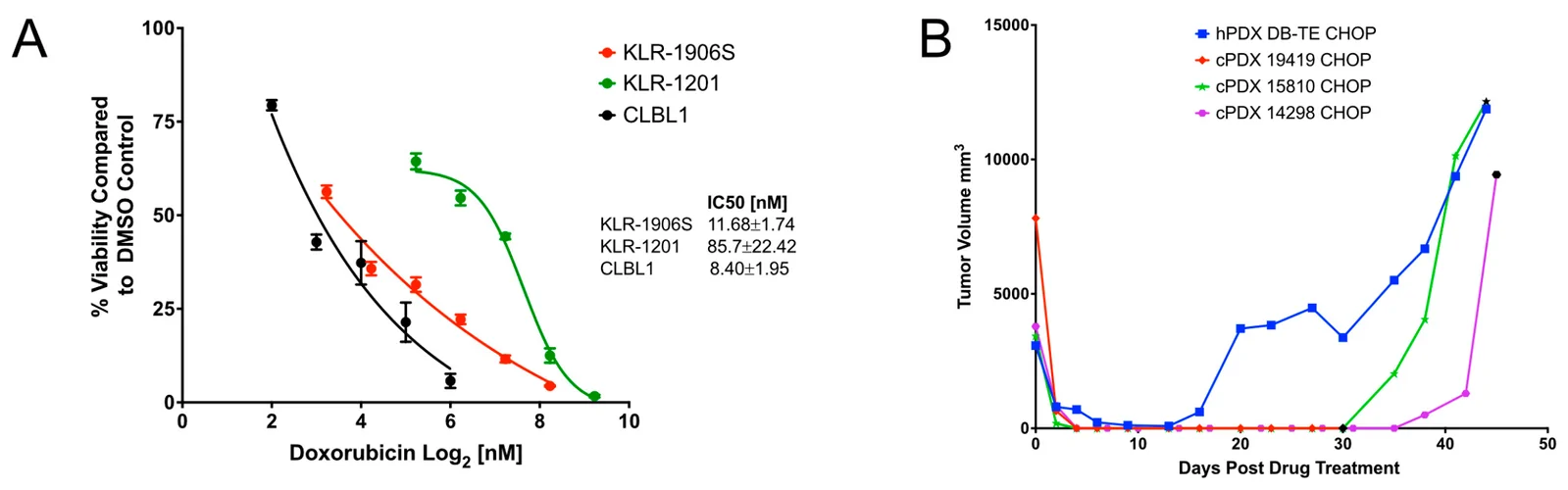
In vitro and in vivo cytotoxic drug response experiments. (**A**) Dose–response curves of cell lines KLR-1201, KLR-1906S, and CLBL1 treated with doxorubicin. (**B**) Tumor growth curve of 19419, 14298 and 15810, alongside the human DLBCL PDX control DB-TE, xenografted into individual mice and treated with a single course of CHOP.

Canine BCL and human DLBCL are treated with a chemotherapeutic regimen that contains the anthracycline doxorubicin. To predict the response of tumors to chemotherapy, we exposed cell lines KLR-1201 and KLR-1906S to doxorubicin for 48 h. The inhibitory concentration (IC50) of doxorubicin in KLR-1906S was 12 nM, similar to that for the established canine cell line CLBL1, whereas the IC50 for KLR-1201 was 86 nM (Figure 6A). In a proof-of-concept pilot study to assess the potential utility of the new developed PDX for assessing therapeutic responses in vivo, mice bearing the three different cBCL PDXs were treated with CHOP, and complete tumor regression was observed by visual inspection (Figure 6B). However, the tumor recurred in mice with PDX 15810 and 14298, similar to the response observed for the human DLBCL PDX DLBCL_p3_NY-DB-TE. No detectable tumor was observed in 19419 at the time of endpoint. These pilot data suggest that the cBCL PDX will be useful for evaluating the efficacy of therapeutics in fully powered future studies.

## 4. Discussion

In this study, we addressed a critical bottleneck in comparative lymphoma biology research by establishing and characterizing novel PDX and cell line models for canine B-cell lymphoma, providing a valuable new resource to advance lymphoma research. We demonstrated that PDX derived from primary lymphoma from canine patients maintained several of the histopathological and immuno-phenotypical features of the original tumors, while also retaining stable overall gene expression profiles and distinct clonal IGHV repertoires of the primary cancers. Furthermore, SNP genotyping and identity by descent analysis showed high concordance between primary tumors, xenografts, and xenograft-derived cell lines (Table 3), a finding that excludes cross-contamination and confirms the genetic fidelity of these models over time.

Establishing and testing different models of canine lymphoma is essential to continue to expand our understanding of the disease, not only to improve outcomes for dogs diagnosed with this cancer but also to continue investigations into the comparative aspect of the human counterpart. A comprehensive biological and molecular characterization of canine lymphoma PDX, as described here, provides us with evidence that they are useful tools for further study. Further, therapeutic targets can be studied by gene suppression or deletion, and validated by in vitro and in vivo testing. We were also able to successfully generate independent cell lines from these PDX tumors, which maintained genetic similarity to the primary tumor. Historically, the generation of cBCL cell lines that do not require stimulation or genetic manipulation to maintain their viability has proven to be quite challenging, hence the limited number of available cell lines available for exploratory research.

While gene expression features related to the primary tumor cells were largely conserved throughout PDX passages, notable changes compared to the primary tumor were largely related to interactions with the microenvironment. This is not unexpected given the change in host species from dog to mouse. This does highlight a limitation in PDX with regard to loss of the primary species stromal and immune microenvironment and limits direct study of this interaction. Another finding was the variation in IGHV among the samples, ranging from continued somatic hypermutation to relatively static, which may reflect specific subtypes.

One limitation of this work is that it has a small sample size. The generation of PDX models is time-consuming and expensive. The ability to collect sufficient amounts of fresh tissue samples from the patient can be challenging, particularly since a diagnosis of canine lymphoma can frequently be made via non-invasive fine-needle aspiration cytology with flow cytometric-based immunophenotyping, as opposed to whole-node extirpation and histopathology.

While canine lymphoma recapitulates many features of human DLBCL, progress in the cBCL field has been constrained by a lack of critical reagents compared to human studies, including a lack of preclinical tumor models, cell lines, and the ability to modify those cell lines genetically [41]. Here, we demonstrate that primary cBCL can be successfully propagated in immunodeficient NSG mice, enabling long-term engraftment for potential therapeutic studies and the derivation of stable in vitro cell lines through a reproducible workflow for their generation.

## 5. Conclusions

In this study, we established and characterized three novel cBCL PDX lines and two cell lines derived from the PDX. These models demonstrated genetic profiles, histologic features, and immunophenotypic markers similar to those of the original canine tumors. Overall, the method we present here demonstrates a repeatable procedure for deriving such reagents, while the new lines themselves will be valuable new resources for investigating lymphoma pathogenesis, identifying novel therapeutic targets, and performing preclinical evaluation of new drugs. This work will help accelerate canine lymphoma research and strengthen related comparative oncology, aiming to advance the treatment of both human non-Hodgkin lymphoma and cBCL.

## Figures and Tables

**Table 1:** Clinical data on canine B-cell lymphoma patients. The lymph node excisional biopsies were taken prior to any therapeutic intervention and at the time of routine staging for two of the three dogs. Treatments were either none or standard of care CHOP (cyclophosphamide, doxorubicin, vincristine, and prednisolone) chemotherapy. CR indicates Complete Response. Stage IV indicates the tumor involved peripheral lymph nodes and the liver or spleen or both, whereas Stage V indicates the tumor involved peripheral lymph nodes, liver or spleen or both, and the bone marrow. Substage b indicates that the dog was exhibiting clinical signs of illness, such as lethargy, inappetence, or weight loss.

ID.	Breed	Sex	Age (Years) *	Treatment	Response	Stage
15810	Rottweiler	Male castrated	6.9	None	n/a	Not done
19419	Mixed	Male castrated	10.0	CHOP	CR	Stage Vb
14298	Rottweiler	Male castrated	3.7	CHOP	CR	Stage IVb

* Age at time of lymph node collection.

**Table 2:** Samples that were genotyped from the primary tumor, PDX, and cell lines. 1906SPDX refers to the tumor from mouse 6 of the 19th passage of primary tumor 15810. 1201PDX and 1301PDX refer to mouse 1 of the 12th and 13th passages, respectively, of patient tumor 19419. Likewise, for the cell lines, P49 and P26 refer to the passage number of those cell lines.

Primary Tumor	PDX	Cell Line
15810	15810_1906SPDX	KLR-1906SP49
19419	19419_1201PDX 19419_1301PDX	KLR-1201P26
		1771
		CLBL1

**Table 3:** Pi-hat values for each pair of samples, calculated using 132,757 genome-wide SNPs. Samples included the primary tumor, PDX and cell passages (P26 and P49) from the two cell lines (KLR-1201 and KLR-1906S, respectively) and commercially available cell lines (1771 and CLBL1).

	19419	19419_ 1201pdx	19419_ 1301pdx	KLR- 1201P26	15810	15810_ 1906Spdx	KLR- 1906SP49	1771	CLBL1
19419	-								
19419_1201pdx	0.9978	-							
19419_1301pdx	0.9978	1	-						
KLR-1201P26	0.9973	0.9995	0.9995	-					
15810	0	0	0	0	-				
15810_1906Spdx	0	0	0	0	0.9951	-			
KLR-1906SP49	0	0	0	0	0.9948	0.9997	-		
1771	0	0	0	0	0	0	0	-	
CLBL1	0	0	0	0	0	0	0	0	-

## Data Availability

Genotype data generated for 15810, 19419, 1771, and CLBL1 samples, and the 94 SNPs chosen for cell line fingerprinting are publicly available as binary PLINK files at https://datadryad.org (accessed on 15 March 2023, DOI: 10.5061/dryad.dncjsxmbg). The RNA sequencing data generated in this study have been deposited in the Gene Expression Omnibus (GEO) database under accession number GSE314293. Images of uncropped western blots are provided in Supplemental Figure S3. Cell lines and PDX samples generated in this study are available upon reasonable request through the Cornell Veterinary Biobank (CVB). Requests may be submitted to vetbiobank@cornell.edu and are subject to material availability and applicable approvals.

## Supplementary Materials

The following supporting information can be downloaded at: https://www.mdpi.com/article/10.3390/cells15181684/s1, Supplemental Table S1: Pairwise pi-hat values calculated using the 94 fingerprint SNPs; Supplemental Figure S1: Heatmap created using the 94 cell-line fingerprinting SNPs; Supplemental Figure S2: Validation of CRISPR transduction, gene knockdown and knockout in cell line KLR-1201. Supplemental Figure S3: Images of uncropped immunoblots used to generate Supplemental Figure S2.

## Abbreviations

Abbreviations	Full Term
R-CHOP	Rituximab, cyclophosphamide, doxorubicin, vincristine, prednisone
NHL	Non-Hodgkin lymphoma
IHC	Immunohistochemical
IGH	Immunoglobulin heavy chain
IGHV	Immunoglobulin heavy chain variable region
GCB	Germinal center B-cell like
ABC	Activated B-cell like
TRAF3	TNF receptor-associated factor
cBCL	Canine B cell lymphoma
SETD2	SET domain containing 2
NSG	NOD scid gamma
SNPs	Single-nucleotide polymorphisms
MAF	Minor allele frequency
IBD	Identity by descent
FBS	Fetal bovine serum
iDEP	Integrated Differential Expression & Pathway analysis
FNA	Fine needle aspiration
MCM6	Maintenance complex component 6
PLK4	Polo-like kinase 4
