# Supplementary material for: Establishment of Patient-Derived Xenotransplant and Cell Line Models for Canine B-Cell Lymphoma
Source: Cells. 2026 Sep 17;15(18):1684. doi: 10.3390/cells15181684 (PMC13605947; doi:10.3390/cells15181684)
Supplement: Supplementary file 1 [file cells-15-01684-s001.zip › cells-4535917-supplementary.pdf]

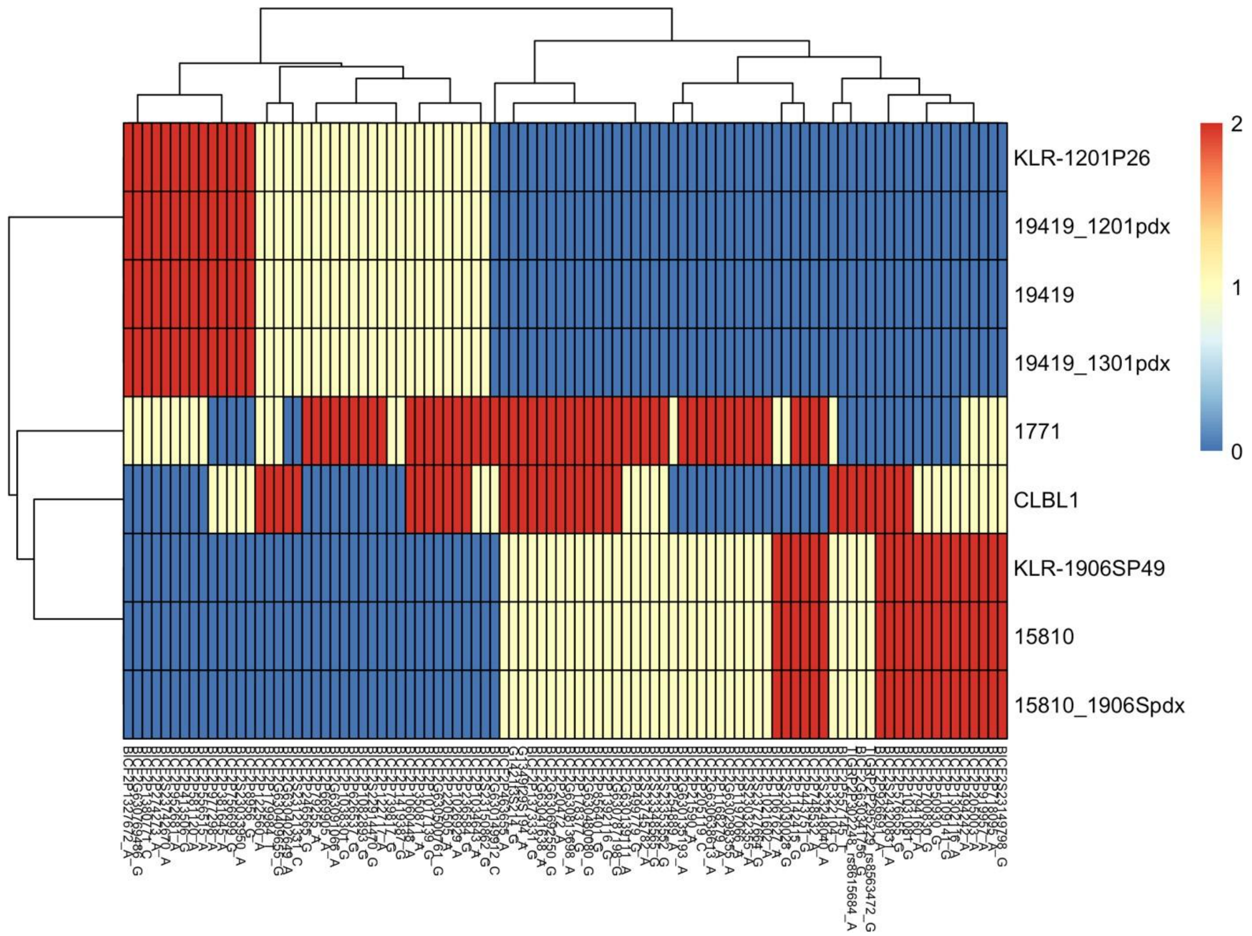

Supplemental Figure S1. Heatmap was created using the 94 cell-line fingerprinting SNPs to show the relationship between the nine samples genotyped in this study. Colors represent 0 (blue), 1 (yellow) or 2 (red) copies of the minor allele for each SNP.

15810	19419	1771	CLBL1
0.5456	0.2404	0.4499	0.5336
0.5217	0.2178	0.3423	0.4499
0.5097	0.1815	0.3423	0.4445
0.5000	0.1492	0.3303	0.4260
0.4738	0.1256	0.2944	0.4260

Supplemental Table S1. Pairwise pi-hat values calculated using the 94 fingerprint SNPs, comparing two of the primary cBCL in the present study (15810, 19419), two commercial cell lines (1771, CLBL1) and 5,981 dogs previously genotyped. The five highest pi-hat values are shown for each sample from the present study. Note that the tumor, PDX and cell line samples from each of 15810 and 19419 all had a pairwise pi-hat of 1.000

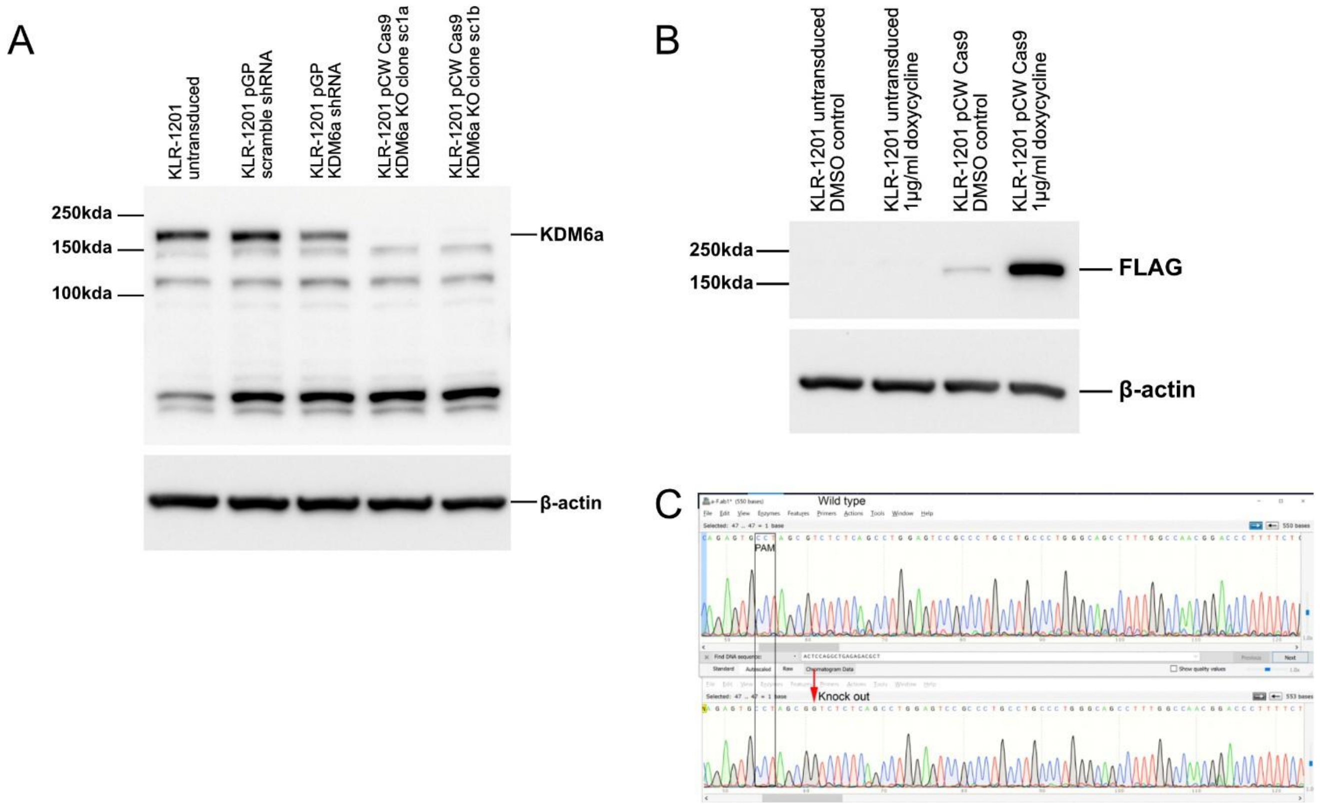

Supplemental Figure S2. Validation of shRNA- and CRISPR-Cas9-mediated depletion of KDM6A in cell line KLR-1201. (A) Western blot showing KDM6A (top) and b-actin (bottom) protein expression in untransduced cells, lower levels with *Kdm6a* targeted shRNA and complete knock out with CRISPR targeted guide following single cell cloning. Lane 1: Marker, Lane 2: Untransduced cells, Lane 3: Scrambled shRNA, Lane 4: KDM6a shRNA. Lane 5: KDM6a KO clone #1. Lane 6: KDM6a KO clone #2. Lane 7: Marker. (B) Western blot for FLAG tagged-Cas9 in KLR-1201, showing FLAG (top) and b-actin (bottom) expression only in transduced cells following doxycycline induction. Lane 1: Marker, Lane 2: Untransduced cells, Lane 3: Untransduced cells, 1ug/ml doxycycline, Lane 4: Cas9 transduced cells, DMSO control. Lane 5: Cas9 transduced cells, 1ug/ml doxycycline. Lane 6: Marker. Lane 7-10 of b-actin WB was from a different experiment. (C) Sanger sequencing results showing insertion of a guanine residue (bottom histogram, red arrow) five residues upstream of the PAM sequence from a single cell clone transduced with Cas9 and *Kdm6a* guide RNA (bottom), resulting in disruption of gene expression, while wild type sequence is maintained in Cas9 transduced cells (top).

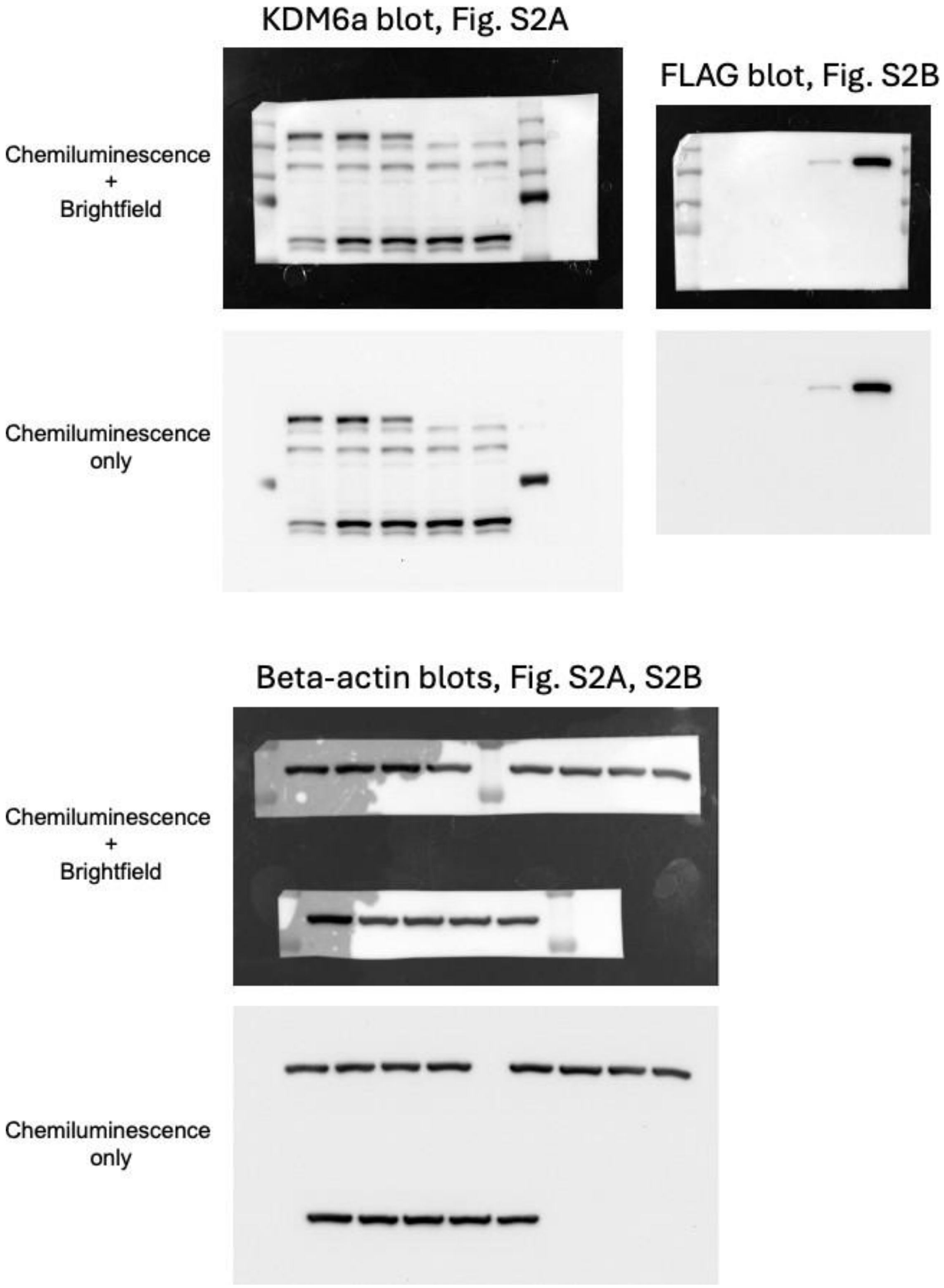

Supplemental Figure S3. Images of uncropped immunoblots used to generate Supplemental Figure S2. For each blot, images of combined chemiluminescence and brightfield signal, or chemiluminescence alone, are shown.
